# Biology and augmentation of tendon-bone insertion repair

**DOI:** 10.1186/1749-799X-5-59

**Published:** 2010-08-21

**Authors:** PPY Lui, P Zhang, KM Chan, L Qin

**Affiliations:** 1 Department of Orthopaedics and Traumatology, Faculty of Medicine, The Chinese University of Hong Kong, Hong Kong SAR, China; 2 The Hong Kong Jockey Club Sports Medicine and Health Sciences Centre, Faculty of Medicine, The Chinese University of Hong Kong, Hong Kong SAR, China; 3 Program of Stem Cell and Regeneration, School of Biomedical Science, The Chinese University of Hong Kong, Hong Kong SAR, China; 4 Translational Medicine Research and Development Center, Institute of Biomedical and Health Engineering, Shenzhen Institutes of Advanced Technology, The Chinese Academy of Science, Shenzhen, Guangdong Province, China

## Abstract

Surgical reattachment of tendon and bone such as in rotator cuff repair, patellar-patella tendon repair and anterior cruciate ligament (ACL) reconstruction often fails due to the failure of regeneration of the specialized tissue ("enthesis") which connects tendon to bone. Tendon-to-bone healing taking place between inhomogenous tissues is a slow process compared to healing within homogenous tissue, such as tendon to tendon or bone to bone healing. Therefore special attention must be paid to augment tendon to bone insertion (TBI) healing. Apart from surgical fixation, biological and biophysical interventions have been studied aiming at regeneration of TBI healing complex, especially the regeneration of interpositioned fibrocartilage and new bone at the healing junction. This paper described the biology and the factors influencing TBI healing using patella-patellar tendon (PPT) healing and tendon graft to bone tunnel healing in ACL reconstruction as examples. Recent development in the improvement of TBI healing and directions for future studies were also reviewed and discussed.

## 1. The Attachment of Tendon to Bone - Tendon-Bone Insertion (TBI)

**Figure 1:**
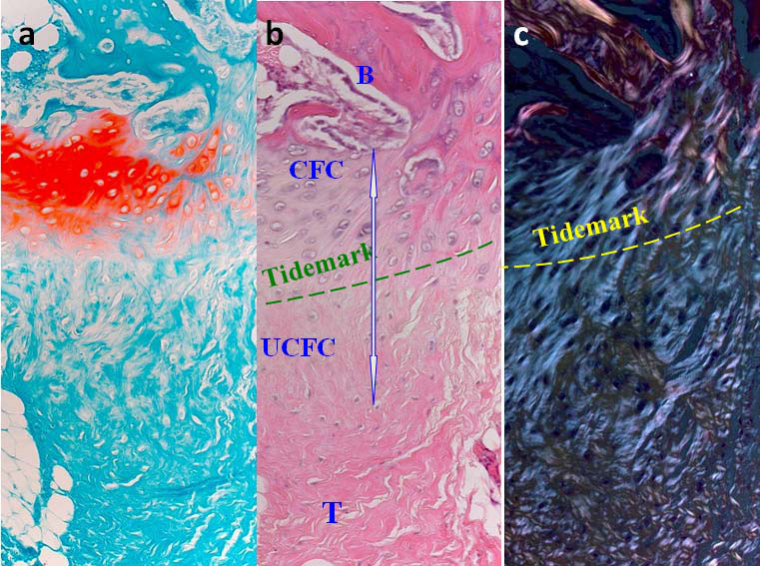
**Photomicrographs showing the (a) Safrainin-O staining; (b) H&E staining and (c) polarized microscopic image of the direct tendon-to-bone insertion**. Note the gradual transition of the four zone at the direct tendon-to-bone insertion. Magnification: 20×; B: bone; CFC: calcified fibrocartilage; UFC: uncalcified fibrocartilage; T: tendon.

The attachment of tendon to bone presents a great challenge in engineering because a soft compliant material (tendon) attaches to a stiff (bone) material[1]. A high level of stress is expected to accumulate at the interface due to the difference in stiffness of the two materials[2]. This problem is solved by the presence of a unique transitional tissue called "enthesis" at the interface which can effectively transfer the stress from tendon to bone and vice versa through its gradual change in structure, composition and mechanical behavior. There are two types of entheses at the tendon to bone insertion (TBI) based on the how the collagen fibers attach to bone[3]. Direct insertions (also called the fibrocartilaginous entheses), such as the insertion of anterior cruciate ligament (ACL), Achilles tendon, patellar tendon, and rotator cuff as well as femoral insertion of medial collateral ligament (MCL), is composed of four zones in order of gradual transition: tendon, uncalcified fibrocartilage, calcified fibrocartilage and bone (Figure 1). The continuous change in tissue composition from tendon to bone is presumed to aid in the efficient transfer of load between the two materials. Current research also indicates that the mineralized interface region exhibited significantly greater compressive mechanical properties than the non-mineralized region[4]. In direct insertions, tendon/ligament fibers are passed directly into the cortex in a small bone surface area. Superficial fibers are inserted into the periosteum, but deep fibers are attached to bone at right angles or tangentially in the transition. Indirect insertions (also called fibrous entheses), such as the tibial insertion of the MCL and the insertion of the deltoid tendon into the humerus, has no fibrocartilage interface. The tendon/ligament passes obliquely along the bone surface and inserts at an acute angle into the periosterum and is connected by Sharpey's fiber over a broader area of tendon and bone[5,6]. Indirect and direct insertions confer different anchorage strength and interface properties at the tendon-bone interface. The main factors affecting the type of insertion seem to be strain, site, length and angle of insertion. When a ligament runs parallel to the bone, as in the MCL, the insertion is more likely to be indirect, while when the ligament enters the bone quite perpendicularly (as in ACL), the insertion is direct. Indirect insertion may be elevated off the bone without cutting the ligament itself, where direct insertion requires cutting the substance of the ligament to detach it[7].

TBI injuries are very common in sports. Surgical reattachment of tendon and bone often fails and presents difficulty for tendon to bone healing due to the lack of regeneration of this specialized structure[8-15]. For example, the failure rates for rotator cuff repair have been reported to range from 20% to 94%[16,17]. Similarly, ACL reconstruction, which requires a tendon graft to be put inside a bone tunnel, has failure rate ranged 10%-25%, depending on the evaluation criteria used[18]. It is hypothesized that poor vasculature at the fibrocartilage zone in the enthesis may contribute to the poor healing response. However, the issue is more complicated as factors like mechanical loading, extracellular matrix composition and biological factors are likely to interact to affect the healing outcome. Better understanding of its natural healing process as well as factors influencing its healing is essential to the improvement of outcome of TBI healing. This paper therefore aimed to review the biology of healing in preclinical animal models as well as the current biological and biophysical treatment modalities for the augmentation of the regeneration of TBI, using direct tendon to bone repair in patellar-patella tendon (PPT) and tendon graft healing inside a bone tunnel in anterior cruciate ligament (ACL) reconstruction as examples.

## 2. Challenges in Different Types of TBI Healing

### 2.1 ACL reconstruction

ACL is an important static stabilizer of the knee. Tears or ruptures of ACL are very common painful injuries, especially in sports medicine. Our previous study showed that 38.5% of male patients who underwent knee arthroscopy following trauma had ACL tears[19]. ACL cannot repair itself when injured. ACL reconstruction is therefore frequently performed in order to restore joint stability and thereby minimize injury to both the chondral surfaces and surrounding tissues. Approximately 95,000 incidences of acute rupture of ACL occur and more than 50,000 knees are reconstructed annually in US[20]. Conventional ACL reconstruction is not a universally successful procedure, with failure rate ranged 10%-25%, depending on the evaluation criteria used[18]. The clinical challenges associated with ACL reconstruction are graft laxity and inferior mechanical properties compared to those of native insertion; unsatisfactory time and protocol for rehabilitation and donor site morbidity.

As ACL has poor healing capacity, reconstruction of ACL with tendon graft is commonly performed. Autologous bone-patellar tendon bone and hamstring grafts are presently the most commonly used grafts for ACL reconstruction, with the use of hamstring tendon autograft becoming more popular given the morbidity induced by using bone-patella tendon-bone autograft. It is important to note that bone-to-bone healing occurs within the tunnels in the bone-patellar tendon bone graft whereas tendon-to-bone healing happens in hamstring graft without bony ends. With the growing popularity of using the hamstring graft for ACL reconstruction, studies on the biology and treatment options for improvement of tendon graft to bone tunnel healing have become the focus of research in ACL reconstruction.

### 2.2 PPT repair

Trauma, overloading or chronic disorder induced injuries to the human patella-patellar tendon complex are not uncommon, such as in patellar fracture, patellar tendon rupture or separation of the patellar tendon from the patella. If injuries involve the patella, the clinical treatment can be fracture repair, partial or even total patellectomy[21,22]. It is well known that the patella is an important functional component of the extensor mechanism of the knee[23]. Therefore, the perceived role of the patella in knee function has profoundly influenced the preferred treatment of injuries to the PPT complex. Since total patellectomy results in permanent dysfunction of the knee with decreased extensor strength, extensor lag, quadriceps atrophy, and ligamentous instability, every effort should be made to preserve as much of the patella as possible and to understand the healing taking place at two different or imhonogenous tissues between patellar tendon and remaining patella. We also demonstrated the inferiority of PPT healing as compared to healing in patellar fracture (bone to bone repair), with no typical intermitted fibrocartilage zone as seen in normal TBI[24].

## 3. Animal models for the study of TBI Healing

### 3.1 ACL reconstruction

**Figure 2:**
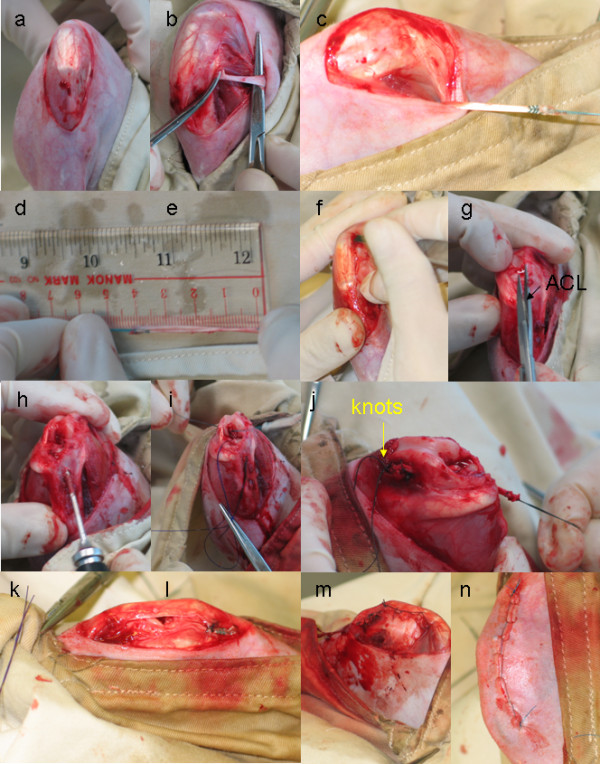
**ACL surgical operation procedures**. (a) Expose knee joint; (b) Isolation of semitendinous graft; (c) Tide graft with holding suture; (d) Record the length and diameter of the graft; (e) Dislocate the parapatellar and remove the fat pad; (f) Identification and dissection of ACL; (g) Drilling of bone tunnel; (h) Pull the tendon graft into the tunnel; (i) Tide the femoral and tibial ends of graft to periosteum with knots at tension at 30° knee flexion; (j) Re-locate parapatellar; (k) Parapetaller wound closure; (l) skin wound closure.

In order to better understand the biology of tendon graft to bone tunnel healing after ACL reconstruction and to develop strategies for the improvement of outcome, animal models are essential. Rabbit, rat, canine and sheep models have been developed and used for the study of natural tendon graft to bone tunnel healing and treatment outcomes. Compared with other animal models, rabbit and sheep models are more commonly used due to their low cost and large size, respectively. Only a few research groups have used rat model due to its small size and hence the difficulty in performing the surgery. Our group has established both the rabbit and rat models[25-30]. Under general anesthesia, the tendon graft is harvested. The ACL is then excised after medial parapatellar arthrotomy. A tibial tunnel and a femoral tunnel with diameter matching the graft diameter are then created from the footprint of the original ACL to the medial side of the tibia or lateral-anterioral femoral condyle, respectively, with an angle of 55° to the articular surface. The tendon graft is then inserted and routed through the bone tunnels, fixed on the femoral and tibial tunnel exits with suture tied over the neighboring periosteum at maximum manual tension at 30° of knee flexion. Soft tissue is then closed in layers (Figure 2). The animals will be allowed to have free cage movement immediately after operation as desired clinically.

### 3.2 PPT repair

Direct tendon to bone healing has been studied in different TBI sites using different animal models, including patella-partellar tendon (PPT), Achilles-calcaneus insertion, and rotator cuff tendon in rats, rabbits, canine and baboons[31-33]. Using a partial patellectomy model in rabbits, we have investigated TBI natural healing extensively in the past years[24,33-35]. The beauty of this model is that the sagital section of PPT provides a unique and internal comparison of healing between tendon-to-bone (patellar tendon to the proximal remaining patella) and tendon-to-cartilage (patellar tendon.to articular cartilage of the proximal patella).

Because of poor healing capacity in TBI and TBI healing is often delayed in both experimental models [36,37] and patients [38], how to accelerate its healing process therefore becomes a focus of our musculoskeletal research, including studies using rotator cuff model in dogs [39] and in rats [40] as well as studies from authors' group where we used partial patellectomy model in both goats [33] and rabbits[41-44]. Apart from testing better fixation protocols, such experimental models provide a useful platform for evaluation of potential biological and biophysical interventions developed for the acceleration and/or enhancement of TBI repair.

## 4. Nature Healing Process and Factors Affecting TBI Healing

### 4.1 ACL Reconstruction

#### 4.1.1 Healing process and factors influencing tendon graft to bone tunnel healing

**Figure 3:**
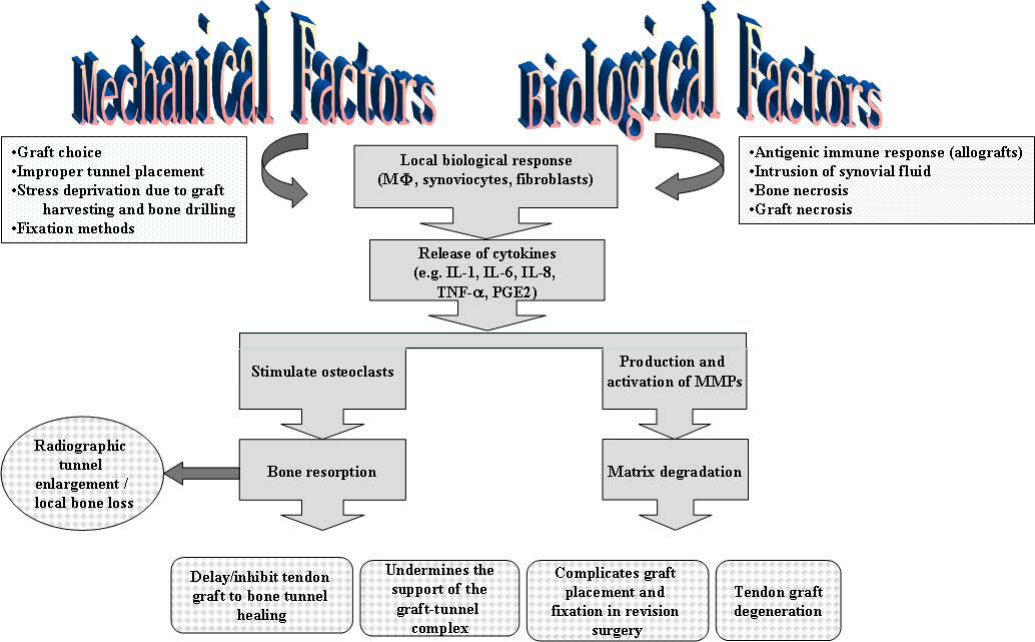
**A schematic diagram showing the contribution of mechanical and biological factors to the sub-optimal healing in ACL reconstruction**.

The tendon graft to bone tunnel site is often seen as the weak link at the early stage of ACL reconstructive surgery. The tendon to bone tunnel complex can achieve only one-tenth of the mechanical strength of native ACL with graft pullout from the bone tunnel at 12 weeks after ACL reconstruction in our rabbit ACL model (Unreported observation). Mechanical and biological factors including graft-tunnel motion, stress deprivation due to graft harvesting and bone drilling, intrusion of synovial fluid after ACL injury, bone necrosis due to trauma, graft necrosis due to avascularity and pressure effect of graft against bone tunnel are the possible factors leading to suboptimal tendon graft to bone tunnel healing in ACL reconstruction. These unfavorable mechanical and biological factors may induce the release of inflammatory cytokines by macrophages, synoviocytes or fibroblasts which may in turns activate osteoclasts for bone resorption and stimulate the production of matrix metalloproteinases (MMPs) for matrix degradation (Figure 3). Understanding the biology of healing is essential to improve the outcome of tendon graft to bone tunnel healing after ACL reconstruction.

Tendon graft to bone tunnel healing can be divided into 4 stages. (1) inflammatory phase; (2) proliferative phase; (3) matrix synthesis and (4) matrix remodeling. During the inflammatory phase, there is an infiltration and recruitment of inflammatory cells and marrow-derived stem cells to the interface. These cells release cytokines and growth factors including TGF-beta and PDGF. There is an ingrowth of blood vessels and nerves as a result of hypoxia or growth factor stimulation[45,46]. The stem cells proliferate and differentiate. During the matrix synthesis phase, MMPs and serine proteases degrade the provisional matrix. The healing cells synthesize and deposit new extracellular matrix with progressive bone ingrowth. At the matrix remodeling phase, the newly-formed bone, interfacial tissue and graft remodel, with establishment of collagen fiber continuity between tendon graft and bone[28,47,48]. The cellularity, vascularity and innervation at the interface decrease. The mechanical strength of the tendon-to-bone tunnel attachment has been shown to correlate with the amount of osseous ingrowth, mineralization, and maturation of healing tissue [25,49], suggesting that bone formation is critical at the early stage of healing. However, bone formation is not the only factor contributing to healing, graft remodeling and graft to bone tunnel integration also affect tendon to bone tunnel healing in addition to bone mass[30].

#### 4.1.2 Types of connection between tendon graft and bone tunnel

**Figure 4:**
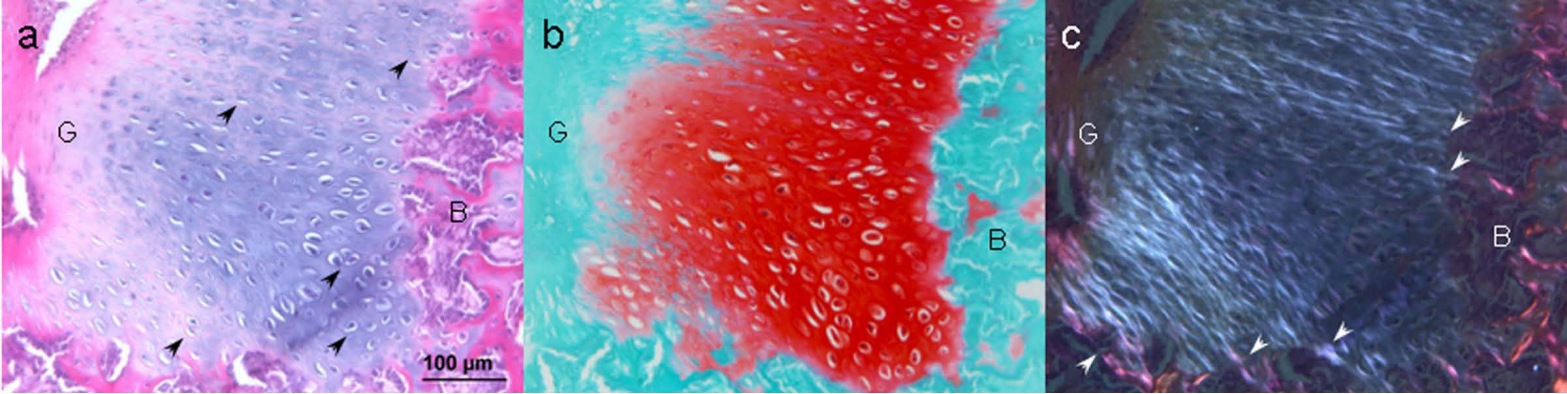
**Photographs showing the presence of chondrocytes at the interface between tendon-bone were associated with better Sharpey's fiber formation and better tendon osteointegration**. (a) H&E staining; (b) SO: Safrainin O staining of corresponding H&E images; (c) Polarized: polarized images of corresponding H&E images of exit segment of femoral tunnel at week 6 after ACL reconstruction in a rabbit model. Magnification: 200×. B: Bone; dark arrowhead: chondrocytes; G: tendon graft; white arrowhead: Sharpey's fibers.

Both direct and indirect insertions between tendon graft and bone have been described in the literature. Some studies have demonstrated the formation of a direct type of insertion with cartilaginous interface between tendon graft and bone, resembling the natural transition zone in ACL[50-54]. The follow up time of the previous animal studies, however, was relatively short and hence the observation of chondrocytes at the interface does not necessary imply the persistence of the fibrocartilage zone as in native ACL. Our result has shown that the chondrocytes functioned as intermediate in endonchondral ossification and disappeared with time during healing and the presence of chondrocytes at the tendon-bone interface was commonly associated with Sharpey's fiber formation and hence better healing (Figure 4)[30]. It has been more widely accepted that the insertion type is an indirect one in which Sharpey fibers secure the junction between the tendon graft and bone[55-58]. Chondrocytes in our study were more commonly observed at the juxta-articular segment of both tunnels at week 12, consistent with the observation of previous studies[59,60]. This was probably due to greater contact stress at the joint level which favored chondrogenesis while shear load occurred inside the bone tunnel[50]. In our study, complete replacement of tendon graft by bone was observed in some regions along the bone tunnel and we believed that this represented the ideal healing inside the bone tunnel[30].

#### 4.1.3 Spatial variation in tendon graft to bone tunnel healing

The healing is not non-uniform at different regions of bone tunnel and at different bone tunnels, with some areas exhibiting better healing than those of the others[30,47,54,60]. Our result has shown that healing at the tibal tunnel was inferior compared to that at the femoral tunnel[28,30], resulting in more frequent pull-outs from the tibial tunnel with bone attachment in rabbit models[28]. The exact reason for inferior healing in tibial tunnel was not clear but we speculated it to be related to the local environment where the tunnel was located. The whole femoral tunnel was located in the cancellous bone while only the juxta-articular segment of tibial tunnel was located in the cancellous bone. Previous study reported better healing with chondrocyte-like cells when the graft was inserted into a cancellous bony tunnel compared to a marrow-filled space[61]. We also observed variation in healing response at different tunnel segments[30]. It has been reported that Sharpey-like fibers were not uniformly present at all sites along[62-64] and around the circumference [50,55,59,62] of the bone tunnel. The reasons for the variation is not clear but alteration of the mechanical and biological environment due to bending of the graft at the aperture, graft micromotion (particularly for suspensory fixation), location of graft in cancellous bony versus a marrow-filled space or intrusion of synovial fluid are possible causes[47]. Because of the variation of healing at different regions of bone tunnel, assessment of healing quality in histology can be very subjective and comparison between studies is difficult due to the lack of a uniform standard. We have established a reliable and valid histological scoring system for the assessment of tendon graft to bone tunnel healing in ACL reconstruction[29]. The histological scoring system allows the comparison of outcomes of different interventional studies and facilitates the interpretation of results of biomechanical test in outcome studies.

#### 4.1.4 Local bone loss after ACL reconstruction

There is no site in human where a tendon or ligament goes into a bone tunnel. The placement of tendon graft inside an artificially created bone tunnel, while providing a large bone surface for tendon graft to bone tunnel healing, also disrupts the physiological mechanical loading, resulting in regional-dependent stress shielding and subsequent bone loss and thereby also negatively impact healing. We reported that there was regional-dependent loss of surrounding trabeculae after ACL reconstruction, with significantly loss at the medial side of femur tunnel as well as posterior and lateral side of tibial tunnel in a rabbit ACL model[27]. Significant BMD loss with only partial recovery several years after operation (up to 10 years) were also reported in clinical studies[65-72]. This occurred despite accelerated rehabilitation and return to previous levels of activity. However, these were not randomized or controlled clinical studies. Bone loss after tendon insertion site injury and repair has also been reported in other animal studies[73-76]. The excessive local bone loss might delay healing. Tunnel widening might occur (our observation) and resulted in a less stable surface for tendon-bone integration. Inflammatory tendon degeneration might occur due to the degradative enzymes produced during bone resorption. All these, if happens, might prevent the incorporation of collagen fibers into the mineralized tissue, favor fibrous tissue formation and compromise graft-tunnel healing (Figure 3)[56,74,76]. Significant bone loss and decreased mechanical properties in the first 21 days after flexor tendon insertion site injury and repair was reported, supporting the relationship between bone loss and strength[73,76]. A recent study also reported a positive correlation between radiographic tunnel widening and postoperative knee laxity[77]. However, the relationship was not causal. Second, bone tunnel resorption could complicate revision surgery (Figure 3). Moreover, it might undermine the support of graft-tunnel complex and result in graft failure even in the ideal case that the graft-tunnel complex heals perfectly (Figure 3).

### 4.2 PPT Repair

Using the PPT rabbit model, we have described the process of direct TBI healing[32]. The healing process consisted of 4 stages: inflammation, scar tissues formation, osteogenesis and its remodeling, and regeneration of fiborcartilage-like-zone[34,43,44,78,79]. Our results consistently suggested that new bone formation and its size predicted the quality of its postoperative healing quality[24,78]. Structurally, we reported that more new bone formed at the patella-patellar tendon healing interface was associated with better regeneration of interpositional fibrocartilage[78]. This is an important bony index for studying the treatment efficacy of potential interventions in vivo or clinically. Whether the findings generated from the PPT healing may also be generalized for radiographic prediction of direct TBI healing quality in regions like Achilles-calcaneus and rotator cuff needs further experimental and clinical investigations.

## 5. Recent Development in the Improvement of TBI Healing

**Figure 5:**
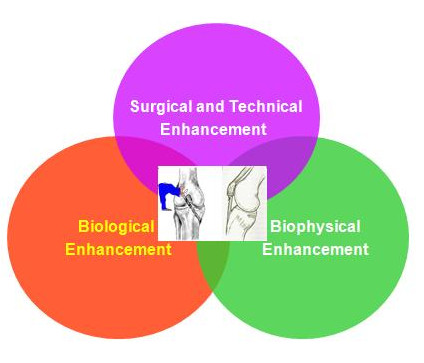
**Approaches for Tendon-Bone Insertion Repair**.

**Figure 6:**
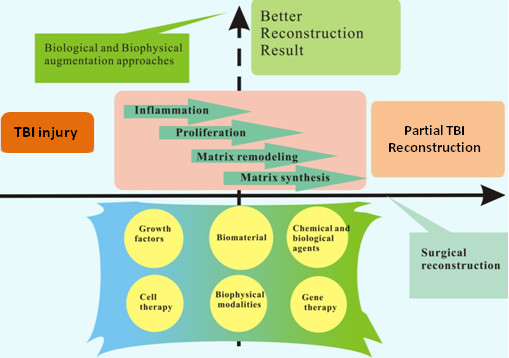
**Diagram summarizing TBI injury treatment options currently available**.

The current treatment and subsequent rehabilitation strategies can be categorized into 3 approaches: surgical or technical, biological and biophysical (Figure 5)[80-83]. A good combination of surgical, biological and biophysical enhancement may improve surgical prognosis and enhance postoperative repair. Figure 6 summarized the current treatment methods for TBI repair based on these 3 approaches.

### 5.1 ACL reconstruction

Mechanical strength of tendon graft to bone tunnel attachment has been demonstrated to correlate with the amount of osseous ingrowth, mineralizaton and maturation of healing tissue[25,49]. Strategies that can increase bone formation and reduce bone loss are being investigated for the improvement of tendon graft to bone tunnel healing. Various methods have been reported to improve healing of tendon graft inside bone tunnel. They can be classified into growth factors, biomaterial, chemical and biological agents, cell therapy, biophysical modalities and gene therapy.

#### 5.1.1 Growth factors

As bone formation is crucial for tendon graft to bone tunnel healing, biological factors such as transforming growth factor-beta1 (TGF-beta1) [84], TGF-beta combined with epithelial growth factor (EGF) [85], recombinant human bone morpohogenetic protein-2 (rhBMP-2) [74,86], bone growth factor [87] and granulocyte colony-stimulating factor [88] have been introduced into tendon graft to bone tunnel interface for the augmentation of healing with good histological and biomechanical outcomes.

#### 5.1.2 Biomaterial

Since calcium phosphate has chemical composition close to bone, there is a recent interest in its use as an osteoconductive material for bone growth. Injectable and solid forms are available. They are primarily for use as bone void filler for the re-contouring of non-weight bearing craniofacial skeletal defects[89]. We have recently reported the augmentation of screw fixation with injectable hydroxylapatite in the weight-bearing region in osteopenic goat[90]. The material was highly osteoconductive, increased the screw pull-out force and energy required to failure when used in screw augmentation. In view of these favorable properties of calcium phosphate, it can be a good candidate for augmentation of healing and hence fixation of tendon inside bone tunnel. The osteoconductive nature of calcium phosphate might also suppress fibrous tissue formation and promote bone ingrowth into the interfacial gap which increased the fixation of tendon inside bone tunnel. Injectable tri-calcium phosphate (TCP) [91], hydroapatite (HA) [92] and brushite calcium phosphate cement, which composted of dicalcium phosphate dehydrate matrix with beta-TCP granules [26], HA powder in collagen gel [93], magnesium-based bone adhesive [94] and hybridization of calcium phosphate onto the tendon graft [54], have been reported to augment grafted tendon to bone tunnel healing.

#### 5.1.3 Chemical and biological agents

Chemical and biological agents acting on different biological processes of tendon graft to bone tunnel healing have been studied for the improvement of healing. After ACL injury [95] and ligament reconstruction [96], matrix metalloproteinases (MMPs) increased in the intraarticular environment, which can adversely affect the healing process. As a result, blockage of MMPs with alpha2-macroglobulin, a plasma glycoprotein and an endogeneous inhibitor of MMPs, has been reported to improve healing of tendon graft in a bone tunnel with more matured interfacial tissue and Sharpey's fibers. The ultimate load to failure was also reported to be significantly greater in the treatment group[57].

It has been reported that macrophages accumulated following tendon-to-bone tunnel repair and might contribute to the formation of a scar-tissue interface rather than to the reformation of a normal insertion site. Based on this finding, liposomal clodronate-induced depletion of macrophage following ACL reconstruction was used and reported to significantly improve the morphologic and biomechanical properties at the healing tendon-bone tunnel interface[97].

As healing of tendon graft in a bone tunnel depends on bone ingrowth into the interface between tendon and bone, excessive osteoclastic activity may contribute to bone resorption, tunnel widening, and impaired healing. In this regards, inhibition of osteoclastic activity by osteoprotegerin (OPG) was reported to increase bone formation around a tendon graft and improve stiffness at the tendon-bone tunnel complex in ACL reconstruction in a rabbit model, while increased osteoclastic activity due to the application of receptor activator of nuclear factor-kappa B ligand (RANKL) impaired bone ingrowth[98].

During graft remodeling after ACL reconstruction, the tendon graft is infiltrated by inflammatory cells and is subjected to ischemic change. Neovascularization occurs during tendon graft to bone tunnel healing. Therefore, tendon graft to bone tunnel healing is expected to improve with neovascularization and shorten ischemic time. Hyperbaric oxygen (HBO) treatment, which has been shown to enhance angiogenesis in various tissues [99-101], was reported to increase neovascularization at the tendon-bone tunnel interface, collagen organization in the tendon graft, tendon osteointegration and the maximal pull-out strength in a rabbit ACL model[102].

#### 5.1.4 Cell therapy

The application of progenitor cells to promote tendon graft to bone tunnel healing has been reported. The implantation of periosteal autograft [103-106], photo-encapsulated rhBMP-2 and periosteal progenitor cells [107], autologous mesenchymal stem cells (MSC) [53,108,109] and synovial MSC [110] and bone marrow aspirates [106] have been reported to accelerate early tendon graft-bone tunnel healing.

#### 5.1.5 Biophysical modalities

Shockwave has been used to improve healing at tendon-bone tunnel interface in rabbits and the effect of shockwave was found to be time-dependent[111]. The exact mechanism of shockwave remains unclear. However, shockwave has been reported to promote bone formation [112], induce neovascularization and improve blood supply at the tendon-bone junction[113,114].

Low-intensity pulsed ultrasound (LIPUS) treatment was also reported to increase the cellular activity at the tendon-bone interface and improved tendon osteo-integration and vascularity in an ovine ACL reconstruction model[115]. Stiffness and peak load of the tendon-bone tunnel complex was also reported to improve compared to the control group after LIPUS treatment[115].

#### 5.1.6 Gene therapy

Compared to single application of growth factor protein, delivery of gene to the target tissue has the advantage of sustained and prolonged release of growth factor. In the regards, tendon graft infected with adenovirus-BMP-2 gene has been reported to improve the integration of tendon graft to bone tunnel in an ACL model[116]. Despite the success, safety and regulatory issues need to be solved before introducing a gene transfer modality for treatment in ACL reconstruction clinically.

### 5.2 PPT Repair

#### 5.2.1 Surgical and technical approaches

Apart from non-operative approach for TBI repair via limb immobilization, surgical fixation can provide immediate fixation and provide better treatment prognosis. In contrast to fracture fixation which fixes two or more bony fragments, TBI repair needs different sutures and fixation techniques to meet local anatomical and functional demands as biomechanical function of TBI at various skeletal sites varies and there is no standard surgical protocol to follow. Therefore, preclinical and clinical studies are required to make surgical recommendations[117]. For example, Klinger and colleagues [82] compared the time-dependent biomechanical properties of the traditional open transosseous suture technique and modified Mason-Allen stitches (group 1) versus the double-loaded suture anchors technique and so-called arthroscopic Mason-Allen stitches (group 2) in rotator cuff repair in adult female sheep. This in vivo study showed that, postoperatively, the group 2 technique provided superior stability and after healing would gain strength comparable to the group 1 technique.

#### 5.2.2 Biological agents

Cytokines play an important role in cell chemotaxis, proliferation, matrix synthesis, and cell differentiation and has been reported to improve TBI healing. The effect of various cytokines and osteoinductive growth factors, such as BMP-2, BMP-7, or rhBMP-12, TGF-beta1, TGF-beta2, TGF-beta3, and fibroblast growth factor, have been tested for TBI healing enhancement. The available data suggested that they were able to improve formation of new bone and fibrocartilage at the healing TBI site structurally and functionally[86,118]. Platelets-related products that contain various growth factors have been reported to promote TBI repair[81,118,119]. Besides endogenous growth factors, exogenous osteopromotive factors, such as phytoestrogenic herbal compounds may also have promotive effect for TBI healing as some of them have both angiogenic and osteogenic effects [120], suggesting that the osteopromotive formula of Traditional Chinese Medicine (TCM) or herbal medicine should be further explored for their potentials in promoting TBI healing and their associated underlying mechanisms.

#### 5.2.3 Biomaterial and cell therapy

A major focus in this area is the development of tissue engineered bone and soft tissue grafts with biomimetic functionality to allow for their translation to the clinical setting. Simple approaches, such as polyglycolic acid sheet has been tested for enhancing TBI repair and regeneration[121]. One of the most significant challenges of this endeavor is promoting the biological fixation of these grafts with each other as well as the implant site. Such fixation requires strategic biomimicy to be incorporated into the scaffold design in order to re-establish the critical structure-function relationship of the native soft tissue to bone interface. The integration of distinct tissue types in TBI necessitates a multi-phased or stratified scaffold with distinct yet continuous tissue regions accompanied by a gradient of mechanical properties[122]. Using the partial patellectomy rabbit model, we have demonstrated that cartilage to tendon healing was superior to tendon-to-bone healing at the early healing stage with collagen fibers across the healing interface[34,41]. It is therefore reasonable to believe that the earlier fusion of cartilage to tendon at the insertion might provide earlier stability along the entire PPT healing complex. Indeed, the interposition of autologous articular cartilage improved the transition zone regeneration in TBI healing in our established partial patellectomy model in rabbits[13]. Despite the promising findings in this study, the use of autologous articular cartilage can lead to donor site morbidity. Therefore, we have engineered an allogenic chondrocyte pellet for reconstruction of fibrocartilage zone at TBI[14,123]. Despite the improvement in TBI healing with the allogenic chondrocyte pellet, much remains unknown about the basic, translational and clinical application of this technique. For example, what are the signaling mechanisms for transforming the hyaline-like cartilage to fibrocartilage after the transplantation of the allogenic chondrocyte pellet? What are the long-term effects and potential immune responses of allogenic engineered condrocyte pellets as well as the feasibility of generalizing the scientific findings for clinical practice because of high-demands on both good manufacturer practice (GMP) and good clinical practice (GCP) even before obtaining FDA or SFDA approval for wide clinical applications?

#### 5.2.4 Biophyisical modalities

Due to high costs of biological approaches and the difficulties in their controllable delivery, biophysical modalities have been tested and widely applied in clinical settings, such as mechanical stimulation, electrical stimulation, pulsed electrical magnetic fields (PEMFs), and LIPUS (at 100-200 bursts, 1.5-2 MhZ, 30 mW/cm^2 ^) that have been evaluated intensively for their potential for enhancing fracture healing or soft tissue repair; the underlying mechanisms for promoting healing are associated chemical and biological responses due to the mechanical stimulations that are in favor of osteogenesis and angiogenesis[124-127]. Clinically, surgical reattachment of tendon to bone is often followed by a longer period of immobilization. Immobilization-induced problems to musculoskeletal tissues are well known in orthopaedic sports medicine and therefore postoperative rehabilitation programs are highly appreciated. As early motion or direct mechanical stimulation, e.g. tension or cyclic loading via external force onto the healing tissue may impair its healing [127-129], using non-contact 'biomechanical stimulations' would be beneficial for augmentation in early healing phase. LIPUS is such a form of mechanical stimulations, i.e. a noninvasive form of mechanical energy transmitted transcutaneously as high frequency acoustical pressure waves in biologic tissues and thus provides a direct mechanical effect on endochondral ossification, osteoblasts proliferation to produce bone by modulating various biosynthesis processes, including angiogenesis[35,130,131]. LIPUS has been documented as a non-invasive mean for accelerating fracture healing, delayed union, non-union, and soft tissue repair process [43,79,126,130,131] as well as promotion of bone mineralization and its remodeling during distraction osteogenesis[132]. The authors of this review paper pioneered in the experimental work for potential clinical indication of LIPUS for accelerating TBI repair and confirmed that LIPUS was generally capable of promoting maturation of inhomogenous tissues, as evidenced with increase in the matrix hardness of the healing tissues at TBI, including new bone, regenerated fibrocartilage and tendon tissues [43], especially with significant augmentation in new bone formation and its remodeling[78]. Similar to soft tissue healing [133], more profound treatment effects were demonstrated in the early healing phase in our series of LIPUS investigation for accelerating TBI repair[42]. Our recent microarray study demonstrated that over 100 genes were related to the underlying molecular mechanism of LIPUS that LIPUS regulated the transient expression of numerous critical genes, especially the cytoskeleton genes in osteoblastic cells[134]. These in vitro results provided further understanding about the role of LIPUS in the regulation of osteoblastic activity potentially involved in osteogensis in TBI repair[134]. A new and interesting finding of this study was up-regulation of genes associated with cell apoptosis, such as BCL2-associated × protein (BAX), suggesting LIPUS accelerated tissue remodeling by activating apoptotic genes and osteogenesis. Our preclinical findings are appreciated by clinicians and patients. The impact of the research findings of LIPUS for TBI repair can be seen from a personal communication with American LIPUS scientists (Dr. Neil Pounder, Smith & Nephew, personal communication) "American surgeons prescribe LIPUS for many patients now, even if FDA only allows the application on non-unions and tibial fresh simple fracture. The surgeons prescribe on other sites at their own risk. One prescription is on Achilles tendon junction healing. But the patients need to claim insurance, where your paper is the key evidence for them to claim the insurance". This is a big contribution to the improvement of patient care. However, not all patients may benefit from such findings. Delayed TBI healing was observed in some patients even after treatment with LIPUS during postoperative examinations in our orthopaedic clinics[135]. For the management of delayed healing in patients with TBI surgery, we tested if extracorporeal shockwave (ESW), which is often used for the treatment of delayed union or non-union [127], would be able to promote TBI repair using a recently established delayed TBI healing model in rabbits[37]. Our findings showed that ESW was able to treat delayed TBI injury by triggering osteogenesis, regeneration of fibrocartilage zone, and remodeling in the delayed TBI animal model[136]. Our preclinical data published in the American Journal of Sports Medicine in February issue of 2008 attracted media's great attention and was reported in *Reuters Health in *New York of USA, with hope of attracting potential clinical applications of ESW in the management of this difficult delayed TBI injury.

Apart from structural restoration of TBI, postoperative functional rehabilitation programs are also essential to achieve full recovery. Exercise program is one of the postoperative rehabilitation programs that help to generate tension to TBI via muscle contraction (concentric force) or passive resistance training (eccentric force). The postoperative programmed FES-induced muscle tension was beneficial for acceleration of TBI repair and was therefore recommended for clinical trials in orthopaedic sports medicine and rehabilitation[44,127]. Although the majority of biophysical intervention studies reported positive results, the forms of biophysical stimulation, its dose effect and application timing shall be further carefully determined.

## 6. Research Challenge and Prospect

Regeneration of the TBI is difficult after injury. This paper described the biology and the factors influencing direct tendon to bone healing using direct attachment of patella-patellar tendon and the tendon graft healing to bone tunnel in ACL reconstruction as examples. Recent work by our group and others in the improvement of tendon to bone healing was also discussed. Despite active research in the understanding of the healing process at the TBI, our understanding is still very limited. Firstly, the origin and maturity of TBI, especially the interpositional fibrocartilage layer have not been clarified. Secondly, the role of different growth factors, mechanical loading and extracellular matrix on natural TBI healing is still not clear. Thirdly, while various biological and biophysical approaches have been demonstrated to be effective for the improvement of TBI healing, the optimal dosage, timing and the underlying mechanisms remain for further investigations. For ACL reconstruction, despite the improvement in tendon graft to bone tunnel healing with different treatment modalities, the mechanical properties of the femur-tendon graft-tibia complex was still inferior to that of the normal ACL and the ultimate failure load can only reach 10-20% that of intact ligament-bone complex in animal studies although it should be noted that ultimate load is also determined by graft mid-substance remodeling besides the tendon graft to bone tunnel healing. Are we able to jump over this hurdle and achieve a higher ultimate load? Will the combination of different strategies give better results? Can we completely replace the tendon graft inside the bone tunnel by bone and recreate the normal tendon-bone insertion at the intraarticualr tunnel exit in ACL reconstruction? Much research needs to be done to improve our understanding and hence the outcome of TBI healing.

## Competing interests

The authors declare that they have no competing interests.

## Authors' contributions

PPYL and KMC prepared the review on tendon graft to bone tunnel healing in ACL reconstruction.

PZ and LQ prepared the review on direct patella-patella tendon repair

## Authors' information

LPPY is currently the assistant professor in the Department of Orthopaedics and Traumatology, The Chinese University of Hong Kong, Hong Kong SAR, China.

ZP is an Assistant Professor and Director Aassistant of Translational Medicine Research & Development Center, Institute of Biomedical and Health Engineering, Shenzhen Institutes of Advanced Technology, Chinese Academy of Sciences, in Shenzhen, China.

QL is the Professor and Director of Musculoskeletal Research Laboratory, Department of Orthopaedics & Traumatology, The Chinese University of Hong Kong in Hong Kong, Hong Kong SAR, China and Director of Translational Medicine Research & Development Center, Institute of Biomedical and Health Engineering, Shenzhen Institutes of Advanced Technology, Chinese Academy of Sciences, in Shenzhen, China.

CKM is the Professor and Chief of Service, Department of Orthopaedics & Traumatology, The Chinese University of Hong Kong, Hong Kong SAR, China.
